# A Rational Fabrication Method for Low Switching-Temperature VO_2_

**DOI:** 10.3390/nano11010212

**Published:** 2021-01-15

**Authors:** László Pósa, György Molnár, Benjamin Kalas, Zsófia Baji, Zsolt Czigány, Péter Petrik, János Volk

**Affiliations:** 1 Centre for Energy Research, Institute of Technical Physics and Materials Science, Konkoly-Thege M. út 29–33, 1121 Budapest, Hungary; posa.laszlo@energia.mta.hu (L.P.); molnar.gyorgy@energia.mta.hu (G.M.); kalas.benjamin@energia.mta.hu (B.K.); baji.zsofia@energia.mta.hu (Z.B.); czigany.zsolt@energia.mta.hu (Z.C.); petrik.peter@energia.mta.hu (P.P.); 2 Department of Physics, Budapest University of Technology and Economics, Budafoki út 8, 1111 Budapest, Hungary

**Keywords:** phase transition, thermal oxidation, thermochromism

## Abstract

Due to its remarkable switching effect in electrical and optical properties, VO${}_{2}$ is a promising material for several applications. However, the stoichiometry control of multivalent vanadium oxides, especially with a rational deposition technique, is still challenging. Here, we propose and optimize a simple fabrication method for VO${}_{2}$ rich layers by the oxidation of metallic vanadium in atmospheric air. It was shown that a sufficiently broad annealing time window of 3.0–3.5 h can be obtained at an optimal oxidation temperature of 400 °C. The presence of VO${}_{2}$ was detected by selected area diffraction in a transmission electron microscope. According to the temperature dependent electrical measurements, the resistance contrast (R_30 °C_/R_100 °C_) varied between 44 and 68, whereas the optical switching was confirmed using in situ spectroscopic ellipsometric measurement by monitoring the complex refractive indices. The obtained phase transition temperature, both for the electrical resistance and for the ellipsometric angles, was found to be 49 ± 7 °C, i.e., significantly lower than that of the bulk VO_2_ of 68 ± 6 °C.

## 1. Introduction

Vanadium is a transition-metal which can coordinate to oxygen in different polyhedral structures forming a large variety of crystalline structures. Besides the single valence vanadium oxides, such as VO, V${}_{2}$O${}_{3}$, VO${}_{2}$ and V${}_{2}$O${}_{5}$, several mixed valence states exist which can be grouped into Magneli series (V${}_{n}$O${}_{2n-1}$) between VO${}_{2}$ and V${}_{2}$O${}_{5}$ phases and Wadsley series (V${}_{n}$O${}_{2n+1}$) between V${}_{2}$O${}_{3}$ and VO_2_ phases. Vanadium-oxides are known by their wide range of applications from catalyst to energy storage [1,2,3]; however, one of their most remarkable features is the semiconductor to metal transition (SMT), due to external stimuli, i.e., temperature or electric field. The electrical conductivity of many vanadium-oxides changes several orders of magnitude as the transition temperature is crossed.

Among the series of oxides, VO_2_ is the most studied material due to its transition close to room temperature at 68 °C, where the crystalline structure of the material reorganises from monoclinic to tetragonal rutile structure [4]. In bulk VO${}_{2}$ besides the five orders of magnitude change in the electrical conductivity, the optical transmission also undergoes a substantial reduction, especially in the near-infrared regime. Therefore, all the optical and electrical properties of the material can be controlled through the SMT. This quality makes VO${}_{2}$ an excellent material for optical switching [5], THz switch [6], sensors [7,8] or resistive switch [4,9]. Smart thermochromic coating on glass is also a promising application, which exploits the variation of transmission around the transition temperature [10]. The film turns opaque with respect to near-IR, without any extra stimuli or energy consumption. However, slightly lower transition temperature (20–40 °C) would be preferable. Doping of the VO${}_{2}$ layer by W, Mo or Nb can improve this drawback [1]. Other studies demonstrated that smaller grain size [11,12] or stress stemming from substrate effect [13] can also reduce the phase transition temperature, but improvement of their reliability is still desired.

Due to the several oxidation states of vanadium, preparation of VO${}_{2}$ film is highly challenging. Among the numerous thin film deposition techniques, such as evaporation [14], pulsed-laser deposition [15], chemical vapor deposition (CVD) [16] and atomic layer deposition (ALD) [17], the magnetron sputtering [11] is the preferred process due to its simplicity and high controllability. However, all of these methods suffer from narrow process windows, i.e., minor changes in the growth parameters can cause significant degradation in the performance of electrical/optical switching. Recently, significant progress has been achieved in the field of low temperature (250–300 °C) deposition using high-power impulse magnetron sputtering (HiPIMS) [18,19,20]; however, the most conventional methods still require high annealing temperatures (typically >500 °C) to improve the crystallinity and the stoichiometry of the film [21,22]. All these requirements make difficult to prepare highly reliable layers and integrate VO${}_{2}$ into the standard CMOS process flow.

Oxidation of metallic vanadium films by thermal annealing provides a cheap and simple method for preparing vanadium-oxides; however, it also requires a precise control of the parameters to achieve the appropriate phase [23]. Since V${}_{2}$O${}_{5}$ is the thermodynamically most stable stoichiometry, at high O${}_{2}$ partial pressure [24] during the oxidation, the VO${}_{2}$ is only an intermediary phase with many other oxides towards the formation of V${}_{2}$O${}_{5}$. This phenomenon is most pronounced during oxidation in air, which would offer a temptingly simple approach for VO${}_{2}$ synthesis. Therefore, reports on thermal oxidation of vanadium [12,23,25,26,27,28,29] also share the difficulty of a narrow process window, i.e., the pressure, the temperature and the annealing time must be adjusted very precisely to obtain the maximum fraction of crystalline VO${}_{2}$.

The present work focuses on the preparation of VO${}_{2}$ films with thermal oxidation of evaporated vanadium films in air. This method, combined with the measurement of electrical resistance, provides a simple, quick and sensitive optimization procedure. We found that a slightly lower than conventionally applied annealing temperature (400 °C) results in a 30 min wide process window in respect to the oxidation time. Moreover, the result of the oxidation was not sensitive to the initial quality of the metal layer; we got the same switching behavior even if the vanadium film was exposed to air for seven months. Detailed studies conducted on structural and optical properties of the optimized film revealed that the electrical and optical switching properties are maintained in case of moderate VO${}_{2}$ content as well. This preparation approach offers a highly flexible and cost effective method to synthesise vanadium-dioxide films.

## 2. Materials and Methods

Metallic vanadium thin films were deposited on (100)-oriented Si wafers covered by 1.3 μm SiO${}_{2}$. Before loading the samples into the oil free evaporation chamber, their surfaces were cleaned with cc. HNO${}_{3}$ and DI water. Vanadium ingot of 99.5% purity was evaporated using an electron gun. The evaporation rate was between 0.2 and 0.3 nm/s, at a pressure of $3\times 10^{-8}\;$Torr during deposition. The film thickness was controlled by a vibrating quartz crystal. The deposited vanadium thickness was 104±4nm according to the in situ measurement. The post deposition heat treatments were carried out in a tube furnace in air at atmospheric pressure. The annealing temperature (T${}_{a}$) varied between 350 °C and 500 °C and the annealing times ($t_{a}$) were between 1 and 4 h. The annealing temperatures were measured by a small heat capacity NiCr-Ni thermocouple.

The optimization of the annealing parameters was based on temperature dependent resistance measurement between room temperature and 100 °C. The hysteresis properties (resistance contrast, transition temperature, hysteresis width) are greatly affected by the stoichiometry and the structural quality of the deposited film, e.g., grain size, stress, cleanness, which allows us a simple and sensitive characterization of the oxide layers. The resistance was acquired between two gold contacts evaporated onto the oxide surface after the oxidation, whereas the temperature was controlled by a Peltier-modul and measured by Pt1000 temperature sensor on the surface of the silicon wafer. The layer was biased by a data acquisition device, while the current was measured by a current amplifier.

The optimized oxide layers were further investigated in respect to its microstructure and optical properties. High resolution transmission electron microscopy (TEM) observation was performed by a JEOL JEM-3010 HREM instrument.Cross sectional TEM specimens were prepared by ion beam milling using a Technoorg Linda ionmill with 10keV Ar${}^{+}$ ions at an incidence angle of 5 with respect to the surface. In the final period of the milling process, the ion energy was decreased gradually to 0.3keV to minimize ion-induced structural changes in the surface layers.

For characterizing the optical properties of the thin film at various temperatures, a Woollam M2000DI spectroscopic ellipsometer was used in a rotating compensator configuration. The sample was placed on a ceramic sample stage located in a custom-made quartz heating cell. The tube-shaped cell had a diameter of 5cm and a length of 20cm and it was sealed on both ends. The sample was measured prior to SMT and subsequently real time measurement was performed with a time resolution of 3s in the available wavelength range of 265nm to 1690nm. During the real time measurement, a maximum temperature of 100 °C was achieved with a temperature gradient of 7 °C/min. The same gradient with the opposite sign was used for reaching room temperature again after the heating process.

## 3. Results And Discussion

### 3.1. Electrical Properties

**Figure 1:**
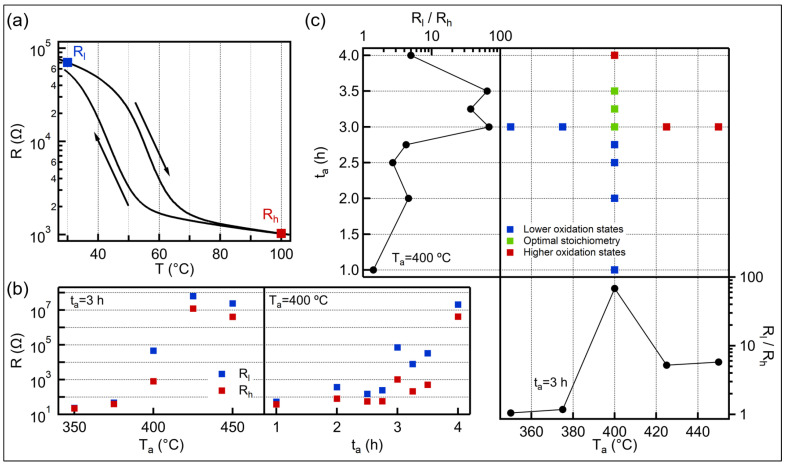
(**a**) Typical temperature dependent resistance curve of VO${}_{x}$ film oxidized at T${}_{a}$ = 400 °C for t${}_{a}$ = 3.0h. The blue/red dot marks the resistance at 30/100 °C on the heating branch (R${}_{l}$ and R${}_{h}$, respectively), whereas the arrows indicate the direction of the hysteresis curve. (**b**) Evolution of the low (blue dots) and high (red dots) temperature resistances as a function of annealing temperature (left panel) and annealing time (right panel), while the other oxidation parameters are fixed. The layers are considered to low oxygen content if the resistance is lower than 1 kΩ and high oxygen content if the resistance is higher than 1 MΩ during the heating cycle. (**c**) The applied annealing time and temperature combinations (middle panel), the colors of the dots indicate the oxidation state of the vanadium according to the electrical property. The resistance switching ratios (R${}_{l}$/R${}_{h}$) are also shown as a function of the annealing temperature (bottom panel) and annealing time (left panel). The films with optimal stoichiometry exhibit good electrical switching effect.

Figure 1a shows a typical temperature dependent electrical resistance trace of an oxidized V film. The nearly two orders of magnitude changes in resistance close to room temperature anticipates VO${}_{2}$ rich content. The quality of the transition is characterized by the three main parameters of the *R*-*T* curve: the transition temperature, the hysteresis width and the magnitude of the resistance change. The transition temperature (T${}_{c}$) is determined by the minimum value of the derivative curve (dlog(R)/dT), is 56 °C during the heating and 43 °C during the cooling branch. The significant lower transition temperature compared to the pure VO${}_{2}$ (68 °C) can be attributed to either the non-stoichiometric composition [30], the stress due to the lattice mismatch with the SiO${}_{2}$ substrate [13] or the small grain size [11,12]. The transition temperature of our VO${}_{x}$ film is about 10 °C lower than layers which were oxidized in air by other groups [23,29,31]. This drop in the T${}_{c}$ may be ascribed to the lower annealing temperature. The hysteresis width (the difference of the heating T${}_{c}$ and cooling T${}_{c}$) is around 10–13 °C, which is a typical value for polycrystalline thin films [23,29]. The magnitude of the transition is calculated by the resistance contrast between 30°C and 100 °C (see blue and red dots, respectively, in Figure 1a on the heating branch) and its value for this particular sample is 68. This resistance switching ratio is in the same regime [23,29,31] or higher [12,28] than the other layers which were prepared by oxidation of metallic V under atmospheric pressure. However, all those samples were annealed at higher temperature and required a more thorough optimization process, because a few percent variation in the oxidation time leads a substantial change in the hysteresis curve.

To examine the effect of annealing parameters to the SMT we varied either the annealing temperature (T${}_{a}$) or the time (t${}_{a}$), while the other parameter was fixed. In left panel of Figure 1b we plot the evolution of low (blue dots) and high (red dots) temperature resistance values as a function of T${}_{a}$ at fixed t${}_{a}$ = 3.0h. Three regions can be observed as we increase the annealing temperature. Below 400 °C, the layers show metallic behaviour with low resistance (<100 Ω). This quality changes suddenly at T${}_{a}$ = 400 °C, where both resistances increase more than one order of magnitude, while the film exhibits resistance switching effect. Finally, above 400 °C, the resistances suddenly increase again, indicating insulator property. This tendency demonstrates well the narrow process window around 400 °C. In contrast, phase transition occurs in a wide range when varying the oxidation time (t${}_{a}$) at a fixed temperature of 400 °C (see right panel of Figure 1b). The transitions between the different oxidation states are also sharp but the quality of the VO${}_{x}$ films does not change significantly between the annealing times of 3.0 and 3.5h. This finding refers to a wide process window, which significantly promotes the reliable production of the VO${}_{2}$ content that contribute to the phase change of the thin films. To demonstrate the robustness of the layer synthesis we created four VO${}_{x}$ films annealed at 400 °C for 3.0h. Between the first and the last oxidation process seven months passed and meanwhile the metal layer was exposed to air, resulting gradually thickening native oxide layer. However, despite the different initial conditions, the electrical switching effect is always presented, whose magnitude varies between 44 and 68 and the transition temperature is in the range of 55–57 °C and 43–45 °C for heating and cooling branch, respectively.

The applied annealing temperature-time combinations during the optimization process are summarized in Figure 1c, whereas the corresponding resistance switching ratios (R${}_{l}$/R${}_{h}$) are shown in side panels as a function of the annealing parameters. The layers are classified into three categories according to their electrical properties. If the switching ratio is higher than 10, the oxide layer is considered to VO${}_{2}$ content (green dots). Those samples that do not show electrical switching are denoted to low/high oxygen content if they show metallic/insulating behaviour (blue and red dots, respectively). We obtain VO${}_{2}$ rich film, when the annealing time is between 3.0–3.5h at T${}_{a}$ = 400 °C. The background of this wide process window in the oxidation time can be explained by exponential dependence on the oxidation rate from the temperature. The reaction rate of the vanadium oxidation can be described by the Arrhenius expression with activation energy between 128–177kJ/mol [23,32]. The optimal annealing temperatures in case of 3.0h and 3.5h annealing time are around 400 °C and there are only a few degrees difference between them [23]. Therefore, the oxidation state must change slowly during this period, opening a wide process window in the oxidation time.

### 3.2. Structural Analysis

**Figure 2:**
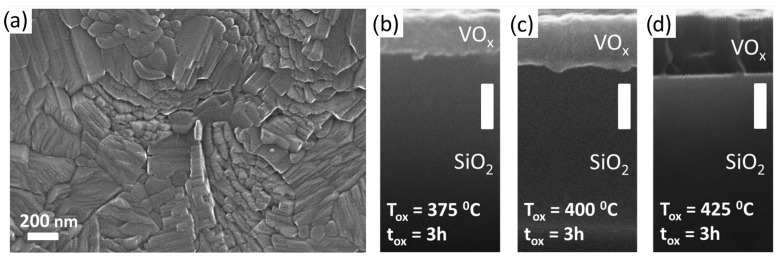
(**a**) Scanning electron microscope (SEM) micrograph of the top surface of a VO${}_{x}$ film oxidized at 400 °C for 3.0 h. (**b**–**d**) Series of SEM images of the cross section of VO${}_{x}$ layers oxidized at different temperatures for 3.0 h. The thickness monotonically increases with the temperature. All white scale bars on the images indicate 200nm.

Figure 2a shows a scanning electron microscope (SEM) image about the surface morphology of a VO${}_{x}$ film annealed at 400 °C for 3.0h. The layer has a polycrystalline structure with anisotropic grains whose lateral size can exceed 100–200 nm, confirming the crystalline structure. The thickness variation of the VO${}_{x}$ film as a function of the oxidation time and temperature was studied by taking cross-sectional SEM images. We found monotonically increasing tendency when the annealing temperature was raised from 375 °C to 425 °C, see Figure 2b–d. This is in accord with the theoretical considerations, since during the oxidation process the mass of the vanadium-oxide film increases with the oxidation state, while the mass density monotonically decreases. Taking into account the molar mass and the density of V and VO${}_{2}$, we anticipate a factor of 2.18 in the film thickness expansion if the V layer transforms into pure VO${}_{2}$. The SEM image yields an expansion ratio of 2.06±0.18 in the case of optimal stoichiometry (400 °C, 3.0h), which is close to the theoretical expectation and agree with the results of other groups [25,33,34]. In contrast, the lower/higher oxidation temperature resulted significantly different ratios (1.64 and 2.54, respectively), referring to different oxidation states. This finding indicates that the in situ thickness measurement could also act as a very simple method to optimize the oxidation parameters. Such a large difference in the thickness could not be observed when the oxidation time was varied between 3.0h and 3.5h, the variation of the thicknesses were within the range of 10%.

**Figure 3:**
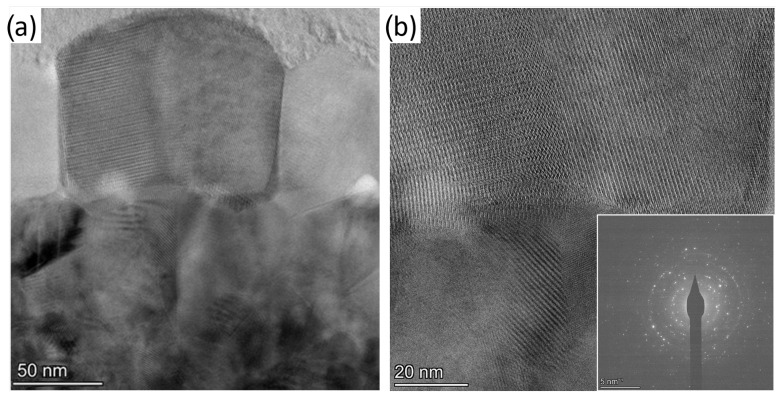
(**a**) TEM images of the VO${}_{x}$ film, showing around 100nm large crytalline particle in the middle. (**b**) High resolution TEM image exhibiting crystalline atomic structure. The inset shows the diffraction pattern of a grain in the VO${}_{x}$ layer.

Figure 3 shows transmission electron microscope (TEM) images of the VO${}_{x}$ film. They reveal that the ≈200nm thick layer is not homogeneous. The bottom part of the film contains smaller particles with a typical grain size of less than 50nm, whereas in the top part, larger grains are presented with lateral sizes of ≈100nm (see Figure 3a). In the high resolution image, the atomic planes are clearly seen in Figure 3b. According to the selected area electron diffraction pattern (see inset of Figure 3b), the crystalline structure is consistent with simultaneous presence of monoclinic and tetragonal phases, which are characteristic to VO${}_{2}$. Although the tetragonal structure should be presented only above the phase transition temperature of the VO${}_{2}$ grains, its existence can be the result of the heating effect of the electron beam. The presence of other oxides, e.g., orthorhombic V${}_{2}$O${}_{5}$ or rhombohedral V${}_{2}$O${}_{3}$ are negligible in the selected area as only a few interference rings can be assigned to these phases. To exclude the effect of the electron beam irradiation, e.g., electron beam-induced crystallization, we monitored the crystalline structure in time, but no changes were observed.

### 3.3. Optical Properties

**Figure 4:**
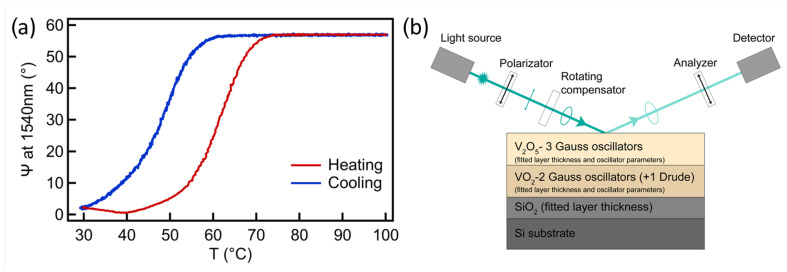
(**a**) Temperature dependent variation of Ψ during the heating cycle at the wavelength of 1540 nm wavelength in the infrared range. (**b**) Schematic of the applied optical model and the SE measurement arrangement.

In order to confirm that a similar low temperature switching occurs also in the near infrared optical properties, an in situ spectroscopic ellipsometry study was carried out. During the temperature dependent spectroscopic ellipsometry (SE) measurement we monitored the complex reflection coefficient (ρ) by collecting the Ψ and Δ ellispometric angles, defined by $\rho =r_{p}/r_{s}=\tan\left(\Psi \right)\cdot \exp\left(i\Delta \right)$, where $r_{p}$ and $r_{s}$ are the complex reflection coefficients of the light polarized parallel and perpendicular to the plane of incidence, respectively. The annealed VO${}_{x}$ layer shows a reversible SMT during the heating cycle (see Figure 4a), the change in the ellipsometric angle Ψ has a maximum around 60° in the infrared wavelength range, in good agreement with previous reports [35]. The parameters of the hysteresis loop are in good accordance with the electrical characterization. The transition temperature (the maximum of dΨ/dT) is around 61 °C during the heating process and 50 °C during the cooling process. The slightly higher transition temperature value in case of SE can be caused that the temperature is measured further from the sample. The optical model was set up according to the cross sectional TEM pictures where two VO${}_{x}$ layers (approximately 100–100nm) were identified with different grain sizes. Thus, the model consists of a semi-infinite Si substrate, a SiO${}_{2}$ layer and two VO${}_{x}$ thin layers. As a result of the analysis, the complex refractive index $\hat{n}=n+ik$ of the top VO${}_{x}$ layer was described by using three Gaussian-oscillators, whereas two oscillators were used for the bottom layer at room temperature and an additional Drude term for describing the metallic behaviour above the transition temperature (see Figure 4b).

The model fit identifies the top VO${}_{x}$ layer as V${}_{2}$O${}_{5}$ phase since the two interband transitions of pentoxide can be clearly seen near 3.0 and 4.5eV [36,37]. The bottom VO${}_{x}$ layer corresponds to the VO${}_{2}$ layer, the optical constants of which at the wavelength of 1540nm at low and high temperature are (n${}_{l}$,k${}_{l}$) = (2.62, 0.47) and (n${}_{h}$,k${}_{h}$) = (2.16, 3.04), respectively, in excellent agreement with a recently reported VO${}_{2}$ film, prepared by oxidation of reactive magnetron sputtered metallic V films [38]. Furthermore, our n${}_{l}$/n${}_{h}$ and k${}_{l}$/k${}_{h}$ ratios are in the ranges reported by other groups in the infrared wavelength range [35,38,39,40].

The topmost V${}_{2}$O${}_{5}$ layer is too thick to be regarded as a native oxide layer, therefore it had to be formed during the annealing process. The thermal oxidation of V film can be described by the Deal–Grove model [41], where diffusion and reaction are the two basic processes. If the initial V film is thick, the diffusion rate is lower than the reaction rate due to the long diffusion distance. In this diffusion-controlled regime the different valence states of V are layered after the oxidation, the highest valence state (V${}^{5+}$, i.e., V${}_{2}$O${}_{5}$) is located at the top of the VO${}_{x}$ layer, while the lowest valence state is located at the substrate-layer interface. Both TEM and SE measurements confirmed the corresponding layered structure and accordingly below the VO${}_{2}$ layer (V${}^{4+}$ valence state), there must be V${}^{3+}$ as well. The V${}^{3+}$ ions play major role in the tuning of the phase transition temperature, the oxygen vacancies reduce the T${}_{c}$ [26]. Our low transition temperature may arise from the high V${}^{3+}$ content. Although the T${}_{c}$ is still too high for smart window application, maybe it can be further reduced by using W doped V layer or V-W alloy. Recently, a new method was introduced, where the transition temperature was reduced to 22 °C by simultaneously sputtering V and W targets [42]. The V${}_{2}$O${}_{5}$ overlayer does not influence significantly the main character of the phase change, similar oxide layer was detected by XPS in other studies [25,26]. However, by etching the V${}_{2}$O${}_{5}$ layer from the surface, the magnitude of the transmission modulation can be increased a little.

Depending on the potential application, different film thickness is desired. In the case of smart window application typically 50–200nm thick VO${}_{2}$ film is applied [10,43], which matches to our layer if only the VO${}_{2}$ layer is considered. However, our optimization process can be generalized to various V layer thicknesses (*d*), in case of diffusion-controlled oxidation, the optimal oxidation time is proportional to the square of the thickness (t${}_{a}∼\;$d${}^{2}$) [44]. The lower limit of the V thickness, i.e., the transition between the reaction and diffusion-controlled oxidation, was found to be around 60 nm [26].

In conclusion, a simple and rational technique was demonstrated to fabricate VO${}_{2}$ coatings. Since the oxidation of metallic vanadium is carried out at atmospheric air at a relatively low annealing temperature (400 °C), it is prosperous for mass production. Moreover, the low temperature phase transition of 49 ± 7 °C makes it a promising candidate as an infrared transmission blocking layer.

## Data Availability

The data is available on reasonable request from the corresponding author (J.V.)
